# Current perspectives of the signaling pathways directing neural crest induction

**DOI:** 10.1007/s00018-012-0991-8

**Published:** 2012-05-01

**Authors:** Timothy J. Stuhlmiller, Martín I. García-Castro

**Affiliations:** Department of Molecular, Cellular, and Developmental Biology, Yale University, PO Box 208103, New Haven, CT 06520-8103 USA

**Keywords:** Neural plate border, FGF, BMP, Wnt, Notch, MAPK, Smad, β-Catenin

## Abstract

The neural crest is a migratory population of embryonic cells with a tremendous potential to differentiate and contribute to nearly every organ system in the adult body. Over the past two decades, an incredible amount of research has given us a reasonable understanding of how these cells are generated. Neural crest induction involves the combinatorial input of multiple signaling pathways and transcription factors, and is thought to occur in two phases from gastrulation to neurulation. In the first phase, FGF and Wnt signaling induce NC progenitors at the border of the neural plate, activating the expression of members of the Msx, Pax, and Zic families, among others. In the second phase, BMP, Wnt, and Notch signaling maintain these progenitors and bring about the expression of definitive NC markers including Snail2, FoxD3, and Sox9/10. In recent years, additional signaling molecules and modulators of these pathways have been uncovered, creating an increasingly complex regulatory network. In this work, we provide a comprehensive review of the major signaling pathways that participate in neural crest induction, with a focus on recent developments and current perspectives. We provide a simplified model of early neural crest development and stress similarities and differences between four major model organisms: *Xenopus*, chick, zebrafish, and mouse.

## Introduction

**Fig. 1:**
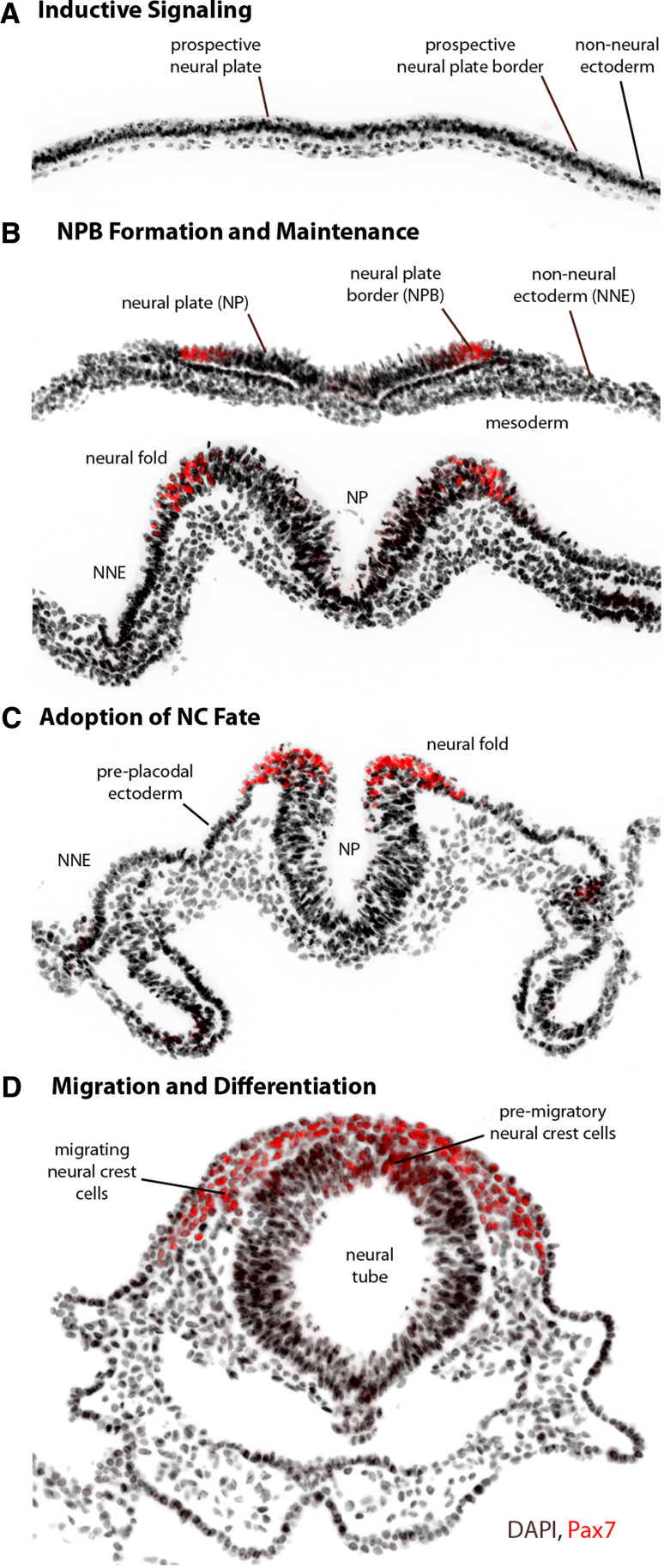
Morphogenesis and major events in early neural crest development. Images display major morphogenetic changes in the early stages of neural crest (NC) development from gastrulation to neurulation, using the chick embryo as an example. The neural plate border (NPB) and neural crest (NC) progenitors are marked by Pax7 in *red*. **a** Signaling molecules induce NC progenitors at the prospective NPB before and during the gastrula stage, but the source of inductive signals varies by organism. **b** NC progenitors are first identifiable with molecular markers of the neural plate border (NPB), including Msx1/2, Pax3/7, Zic1, Dlx3/5, Hairy2, Id3, and Ap2. The NPB is flanked medially by the neural plate (NP) and laterally by the non-neural ectoderm (NNE), with a layer of mesoderm found underneath. At the neurula stage, signaling between these tissues maintains the expression of NPB markers. **c** As the NP thickens and rises, the transcriptional activity of NPB specifiers and additional signaling events lead to the expression of NC specifiers at the neural folds, including Snail2, FoxD3, Sox9/10, Twist, cMyc, and Ap2. The pre-placodal ectoderm is found immediately lateral to the NC in rostral regions. **d** Soon after the NC fate is established, NC cells undergo an epithelial-to-mesenchymal transition and migrate throughout the body and differentiate into a multitude of derivatives. In the chick, NC cells migrate soon after the neural tube fuses, but in most other organisms, NC cells begin to migrate before the neural tube is closed

The neural crest (NC) is a remarkable population of multipotent embryonic cells unique to vertebrates, which migrate from the dorsal neural tube early in development to give rise to a diverse array of derivatives, including neurons and glia of the peripheral nervous system, sympathoadrenal cells, cardiac cells, melanocytes, and most of the bone and cartilage of the face and skull. Their origin can be traced to the border of the neural plate—a region of ectoderm situated between the neural plate (NP), which gives rise to the central nervous system, and the non-neural ectoderm (NNE), which forms the epidermis. Immediately beneath the ectoderm there is a layer of mesoderm, and together with the NP and NNE, these tissues are collectively believed to contribute to the induction of the NC. As the neural plate begins to close to form the neural tube, presumptive NC cells occupy the dorsal tips of the neural plate (the neural folds), and are laterally flanked by prospective placodal ectoderm in cranial regions and by prospective epidermis in the trunk and tail. In all organisms, NC cells undergo an epithelial-to-mesenchymal transition (EMT) in a rostrocaudal wave and take on stereotypical patterns of migration and give rise to various cell types. The rostral, cranial NC cells are the first to delaminate—they begin to migrate before neural tube closure in the mouse, frog (*Xenopus*), and zebrafish, but cranial NC cells in the chick begin migration soon after apposition of the neural folds. At more caudal levels, trunk NC cells migrate from the dorsal aspect of the forming neural tube. The early morphogenesis of NC development is outlined in Fig. 1, using the chick embryo as an example.

NC development is perceived as a step-wise progression from inductive events to transcription factor expression to modulation of migration and differentiation (Fig. 1). The molecules and their interactions have been integrated into a NC gene regulatory network, providing a rich framework for continued functional and comparative research [1, 2]. Initially, the NC is induced by a combination of signals, most notably the bone morphogenetic protein (BMP), fibroblast growth factor (FGF), and Wnt signaling pathways, but also potentially including Notch/Delta, retinoic acid (RA), Hedgehog, and Endothelin signaling. These signaling pathways integrate early in development to induce the expression of a set of regulatory transcription factors (Msx1/2, Pax3/7, Zic1, Dlx3/5, Hairy2, Id3, Ap2), which specify the neural plate border (NPB). These factors along with combinations of the same signaling pathways then trigger the expression of NC specifiers, a second set of transcription factors including Snail2, FoxD3, Sox9/10, Twist, cMyc, and Ap2. NC specifiers are proposed to ultimately control neural crest behavior, from EMT and delamination to migration and differentiation. Because these transcription factors are expressed in other tissues at these and other times in development, their spatiotemporal and combinatorial expression must be considered when associating them with NC development.

Although NC development has been studied in several species, our knowledge of the earliest inductive signaling comes primarily from *Xenopus* and chick research. Recent evidence from these organisms suggests that the NC is induced during gastrulation, and its early development can be explained in a two-step process. An initial phase of FGF and Wnt signaling during gastrulation induces the NC in the prospective NPB, and a second phase of Wnt and BMP signaling during neurulation maintains the NC population. Although the signaling pathways implicated in NC development appear to be conserved among different species, the source, timing, and precise regulation show considerable variation.

The study of early NC development has attracted significant interest owing to the unique properties of these cells. As a great model for induction, pluripotency, cell-fate restrictions, migration and differentiation, NC development involves most elements of developmental biology. Additionally, defects in various aspects of NC development cause a number of debilitating human health conditions, collectively known as neurocristopathies, including aggressive tumors such as melanomas and neuroblastomas, rare syndromes like Hirschsprung and Waardenburg syndromes, and various developmental malformations such as cleft lip/palate and aganglionic megacolon. Therefore, NC biology is of clinical relevance as well, and a fuller understanding of the signaling mechanisms and tissue interactions giving rise to the NC is critical to develop better diagnostic and therapeutic tools for these disorders.

## Timing and transcription factors

**Fig. 2:**
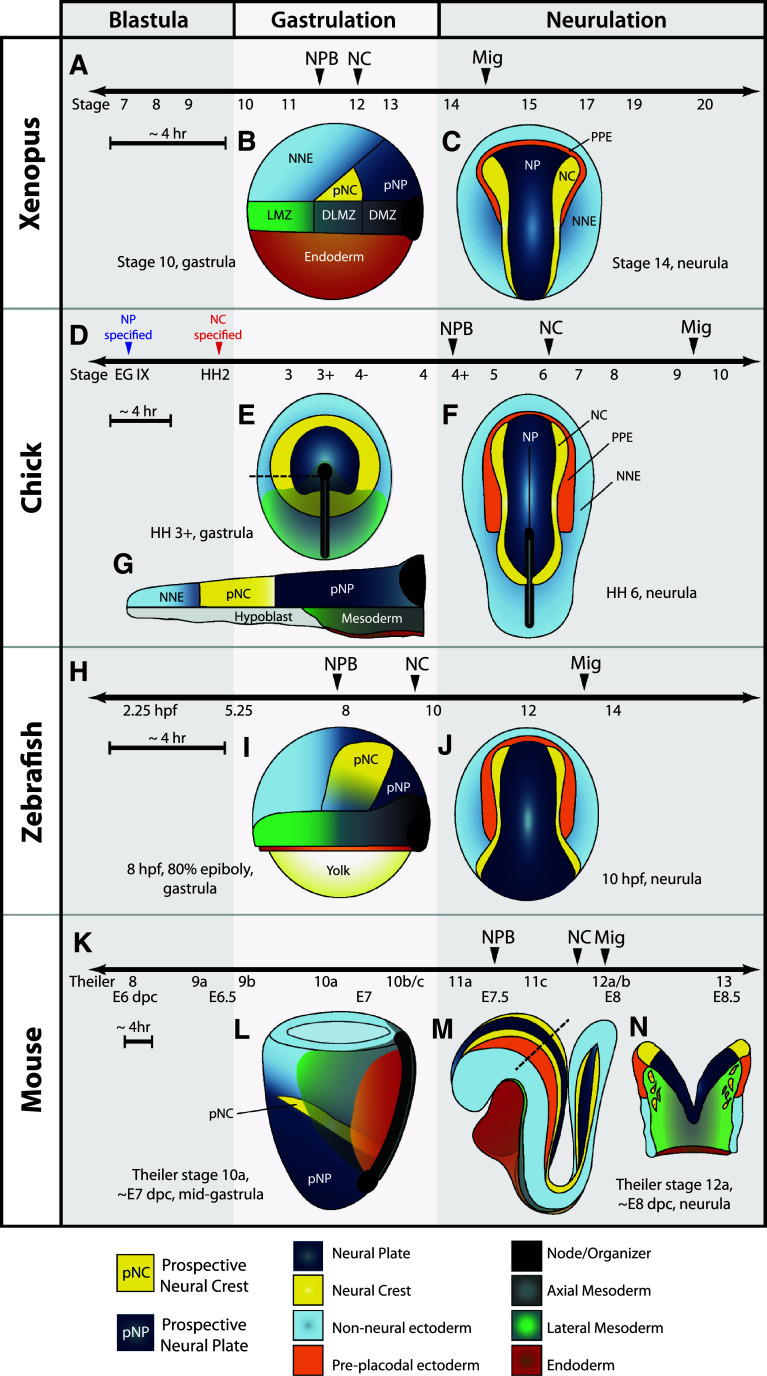
Timing and morphology of early neural crest development in *Xenopus*, chick, zebrafish, and mouse. **a**, **d**, **h**, **k** Timelines for early events in NC development. Note the appearance of neural plate border markers (NPB) and neural crest specifiers (NC) occurs during gastrulation in anamniotes (*Xenopus* and zebrafish) and after gastrulation in amniotes (chick and mouse). Anamniotes progress at a higher rate of development and the time between events is generally very short—compare sizes of ~4-h time bars. **a** In *Xenopus*, markers of the neural plate border are first apparent at Nieuwkoop and Faber stage 11.5 and immediately precede expression of neural crest specifiers at stage 12, before the end of gastrulation. NC migration (Mig) begins around stage 15. **b** Lateral view of early *Xenopus* gastrula. Animal pole is up, dorsal to the right. Prospective neural crest tissue (pNC) is found above the dorsolateral marginal zone (DLMZ), based on fate-mapping studies [31]. *LMZ* lateral marginal zone, *DMZ* dorsal marginal zone. **c** Dorsal view of a *Xenopus* neurula. Anterior is up. **d** In the chick, neural tissue is specified before the egg is laid at Eyal-Giladi (EG) stage IX, while neural crest tissue is specified by Hamburger and Hamilton (HH) stage 2. Markers of the neural plate border are not apparent until after gastrulation at stage 4+. The first neural crest specifiers are not expressed until stage 6. Migration begins between stage 9 and 10. **e** Dorsal view of mid-gastrula. Prospective neural crest tissue is found in a ring around the prospective neural plate (pNP) until post-gastrula stages when the anterior NPB is specified to become pre-placodal ectoderm [30]. **f** Lateral section through the* dotted line* in **e**. At pre-gastrula and early gastrula stages, the prospective neural crest is situated above the hypoblast, an extra-embryonic tissue. As mesoderm and endoderm ingress, the hypoblast is displaced anteriorly, and by the end of gastrulation prospective neural crest tissue is underlain by mesoderm. **g** Dorsal view of neurula, anterior is up. NC specifiers are initially only expressed in the anterior-most aspect of the neural folds. **h** In the zebrafish, neural plate border markers and neural crest specifiers are first expressed during gastrulation. Migration occurs after 13 h post-fertilization (hpf). **i** Lateral view of zebrafish gastrula. Animal pole is up, dorsal to the right. Location of prospective neural crest is inferred from expression of *Msxb* [70] and *Pax3* [113]. **j** Dorsal view of neurula, anterior is up. **k** In the mouse, most neural plate border markers are first detectable around E7.5. Neural crest specifiers are expressed by E7.75, and NC cells begin migrating almost immediately after this expression. Listed below the timeline are approximate stages by Theiler stage, and embryonic days post coitum (dpc). **l** Lateral view of mouse gastrula. Anterior to the left. The mouse embryo develops with the prospective ectoderm as the interior layer. Location of the prospective neural crest is inferred from the position of prospective neural and non-neural tissues, and the expression of NPB markers by E7.5. **m** Lateral view of neurula. Anterior to the left. **n** Section through the* dotted line* in **m**. Although the neural tube has not yet closed, NC cells are migrating extensively

Neural plate border (NPB) specification and neural crest (NC) induction are mediated by a collection of ectodermally expressed regulatory transcription factors from pre-gastrula stages until neurulation. Markers of the NPB are currently the first molecular indication of prospective NC tissue and begin to be expressed during or shortly after gastrulation at a similar timepoint to the appearance of neural tissue (species-specific differences in developmental timing and tissue organization are presented in Fig. 2). Many of the genes involved in NPB formation are expressed in several other tissues and the expression and participation of a given transcription factor can vary between organisms, complicating their analyses. In *Xenopus*, much work has yielded a small set of transcription factors important for NPB specification, but epitasis studies demonstrate that an increasingly complex network exists. Several studies have established Msx1, Pax3, and Zic1 as crucial regulators of NPB specification [3–5], while more recently the participation of Hairy2, Gbx2, Pax7, Ap2a, and Meis3 has also been characterized [6–13]. In the chick, Pax7 is thus far the sole transcription factor implicated in regulating NC specifiers and is expressed exclusively in the NPB at early stages [14, 15]. Other NPB markers are expressed more broadly, with Msx1 and Pax3 additionally expressed more caudal and lateral, Zic genes found more medial, and Ap2 expressed across the lateral NNE [16]. Interestingly, no functional studies have yet confirmed their participation in chick NC induction. In zebrafish, Msx genes are expressed at the border and are involved in NPB specification, but are not necessary for later NC markers [17–19]. Zic2a and Pax3 are expressed more highly in the NP during gastrulation and have not yet been implicated in NPB specification [20]. The expression of Ap2a and FoxD3 overlap in the prospective NC during gastrulation, and their combined activities are necessary for the earliest steps of NC induction [21]. Unique to the zebrafish, Prdm1a (Blimp1) also serves to specify the NPB fate [22–24]. In the mouse, the expression of Ap2 begins as early as E7 with Pax3/7, Msx1/2, and *Zic* genes becoming detectable by E7.5, about the time the neural folds form and slightly before the expression of NC specifiers and the appearance of migratory NCCs ([25–28] and our unpublished observations).

The initial expression of NC specifiers also varies across species. In *Xenopus*, most NC specifiers (Snail2, FoxD3, Sox8/9, others) are first expressed at stage 12, very shortly after the appearance of the NPB, and before gastrulation has even completed [29]. In the chick, however, Snail2 is first evident at stage 6 and not strongly expressed until stage 8 (4-somite stage), several hours after the NPB has formed. Furthermore, definitive NC cells expressing a full complement of NC markers are not apparent until just before migration at stage 9/10 [16]. Despite these differences, the avian NC appears to be specified before gastrulation (having already received the necessary signals for differentiation when cultured in non-inducing conditions) [30], while *Xenopus* neural folds isolated even at the neurula stage do not maintain expression of NC markers without additional signals [31, 32]. In zebrafish, FoxD3 is expressed first and along with Ap2a has a unique role early in gastrulation [21], while Snail2, Sox9/10, and other NC specifiers label the NC towards the end of gastrulation [33]. In the mouse, NC specifiers such as Sox9/10 [34, 35] and FoxD3 [36] label the neural folds very soon after the expression of NPB markers and immediately before cranial NC migration, which is well underway by E8. Additionally, Snail1/2 seem to have switched expression domains in the mouse, but even more intriguing, a double knockout model eliminating both Snail proteins from the epiblast still generates normal, migrating NC cells [37–39]. Snail proteins are thought to be crucial regulators of both NC fate and EMT, and thus, other transcription factors must be providing these essential functions in the double knockout. One could envision redundancy or compensatory mechanisms, and Twist, Zeb1, and Zeb2 have been considered as candidates to provide the lost function. While possible, evidence for significant overlap between the direct targets of Snail proteins and their putative substitutes is currently lacking. Perhaps then, the NC gene regulatory network in the mouse has diverged evolutionarily, dispensing a critical role for Snail genes in murine neural crest development. On this note, knockdown experiments in other organisms generally cause a significant reduction of the NC markers tested, but a demonstration of a complete loss of NC would require a more extensive study, and this should be considered when making comparative analyses between NC gene regulatory networks.

## Evidence for the inductive tissues

NC formation is thought to occur by a classic induction mechanism whereby a tissue or tissues serve as source of inductive signals that are received by another tissue, resulting in the formation of a unique cell type. Pioneering work on NC induction implicated the mesoderm as a potential source of inductive signals—using salamanders, Raven and Kloos were able to generate NC derivatives by grafting presumptive paraxial and lateral plate mesoderm into the naive ectoderm of the ventral blastocoel [40]. Decades later, the inductive capacity of the mesoderm was confirmed in another amphibian, *Xenopus laevis*. *Snail2* was induced in the naive ectoderm of the blastocoel roof following grafts of the organizer or dorsal or lateral mesoderm [41], or by co-culturing explants of naive ectoderm and the paraxial mesoderm [42, 43]. The paraxial mesoderm was subsequently shown to be required for NC formation, as its removal inhibits *Snail2* expression [42, 44].

Other experiments instead suggested that the interaction of neural and non-neural ectoderm led to NC induction. Using two different species of amphibia, Rollhauser-ter Horst demonstrated that neural and epidermal tissues can generate NC cells when juxtaposed [45]. Using pigmented and non-pigmented axolotl embryos in donor/host combinations, Moury and Jacobson later demonstrated that NC cells could arise from both tissues [46]. Similar experiments in *Xenopus* and chick, grafting neural tissue into lateral epidermis, demonstrated both tissues could yield NC cells in these species as well [43, 47]. A recent *Xenopus* paper suggests the competence of the NNE to give rise to NC diminishes towards the end of gastrulation, while the NP retains its competence until neurulation [48]. Together, these findings supported a model where NC induction results from interactions between the NP, NNE, and underlying mesoderm. Recent evidence now suggests that the precise involvement of these tissues is species-specific.

In *Xenopus*, most current models propose that the dorsolateral marginal zone (DLMZ) of the gastrula (which underlies the prospective NC) is the source of NC-inducing signals. The DLMZ expresses multiple Wnt and FGF ligands and the BMP antagonist *Chordin* [31, 41, 49, 50], molecules known to be involved in NC induction. The DLMZ also expresses a number of other Wnt and BMP signaling regulators including Noggin, Cerberus, Frzb1, Dkk1, Sfrp2, and Crescent. A recent study unveils an interesting role for Snail2 in mesoderm formation and implicates this factor in regulating the signals emanating from the DLMZ, making Snail2 crucial for early events in NC development as well [51]. By neurula stages of development, the DLMZ has given rise to the paraxial or intermediate mesoderm, underlying the proper NC. Recombination experiments with the DLMZ and animal caps, or grafts of the paraxial mesoderm into ventral epidermis yield expression of NC markers [31, 32, 50]. Furthermore, explants of the NC at neurula stages do not retain expression of NC markers unless co-cultured with paraxial mesoderm, implicating the mesoderm in the maintenance phase of NC progenitors [31, 32]. A recent study, however, suggests the mesoderm is not necessary for neural or NC induction in *Xenopus*. Injection of an N-terminally truncated form of Cerberus (CerS) inhibits Nodal signaling and mesoderm formation, but still yields expression of *Sox3* (a definitive neural marker in *Xenopus*) and *Snail2* at neurula stages [52]. Although they did not analyze later stages to see if the NC was maintained, this is an intriguing finding. The mesoderm appears to be dispensable for NC induction in zebrafish as well; embryos in which Nodal is inhibited and mutant embryos deficient in Nodal signaling lack mesoderm and mesoderm-derived signaling, but still express all NC markers analyzed [53].

In the avian system, various inductive tissues have been reported. Recombination experiments between nascent neural tissue and paraxial mesoderm from later stages (somites/lateral mesoderm) can yield NC derivatives [47]. Juxtaposition of neural and non-neural ectoderm via grafting approaches in vivo, or via explant co-cultures in vitro also yields NC formation [47, 54]. Yet, NC cells can also be generated from epiblast explants at gastrula stages in the absence of mesodermal and neural markers, and without the addition of exogenous signaling molecules [14, 30, 55]. Thus, while the participation of mesoderm seems to be species-specific, it appears that ectodermal signaling alone may be sufficient to trigger NC induction in the species tested so far. Importantly, there have been no studies regarding the tissue interactions that generate NC in the mouse or any other mammal.

## Major signaling pathways involved in neural crest induction

The molecular era of NC induction was launched in 1993 with studies identifying important effects of FGF2 and Notch in *Xenopus* [56, 57], and Dorsalin1 (a TGF-β molecule) in chick [58]. A series of studies in the following years firmly established the participation of FGF, BMP, and Wnt signaling [41–44, 59–66]. Today, much progress has been gained in understanding the molecular underpinnings of these and other molecules during the earliest stages of NC formation across different species. Here, we summarize current perspectives on the participation of BMP, Wnt, FGF, and Notch signaling pathways in NC induction among the major model organisms: *Xenopus*, chick, zebrafish, and mouse.

### Bone morphogenetic protein signaling

Bone Morphogenetic Proteins (BMPs) are members of the TGF-β superfamily of secreted signaling proteins. BMPs bind to Type I and Type II BMP receptors, and in the canonical pathway lead to activation of Smad1/5/8 proteins. Upon ligand binding, Type I and II receptors form hetero-tetramers, with Type I phosphorylating Smad proteins on their C-terminal domains. Activated Smad1/5/8 proteins then form dimers with Smad4 and translocate to the nucleus and initiate changes in gene expression. BMP receptors are also capable of activating signaling through other, non-canonical pathways such as those mediated by TAK1 (a MAP kinase kinase kinase), but only the canonical Smad1/5/8 pathway is currently known to act in NC induction. Throughout development, BMPs have multiple roles in axial patterning, cell-fate decisions, and left–right asymmetry, and misregulation can lead to cancers (for a review, see [67–69]).

BMP signaling has a crucial role during early development in the establishment of dorsal–ventral polarity and the promotion of epidermal over neural cell fates. The lateral/ventral expression of Bmp ligands and the dorsal/medial expression of BMP antagonists provide the potential to create a gradient of BMP activity. Since the NC forms at the interface between medial and lateral tissues, it was proposed that an intermediate level of BMP signaling is necessary for NC induction. Indeed, epidermal, NC, and neural fates can all be induced in explanted *Xenopus* ectoderm by increasing levels of *Noggin*, supporting the gradient hypothesis [44, 70]. A similar BMP gradient model was proposed in zebrafish [71], and recent evidence suggests a BMP gradient may initially specify the prospective NC domain at the late blastula stage [72]. Another interesting study suggests BMP patterns the ectoderm from anterior to posterior progressively during gastrulation [73]. Although the BMP antagonists Chordin, Noggin, and Follistatin secreted from the organizer and dorsal mesoderm are crucial to establishing a gradient, they may not be necessary for NC development in all organisms. In *Xenopus*, Chordin morpholinos targeted to the DLMZ cause a loss of *Snail2* expression in conjugates with animal caps [31], but in zebrafish, morpholino knockdown of all three BMP antagonists still yields a small domain of NC [53]. Interestingly, double-homozygous null mouse mutants for *Chordin* and *Noggin* actually present increased expression of NC markers *Msx2*, *Ap2*, and *Sox10* at early stages. Later NC populations are expanded and undergo precocious migration, suggesting that BMP antagonists in the mouse actually may serve to suppress NC development [74]. Thus, BMP activity might be modulated to levels permissible for NC induction by other mechanisms in addition to or apart from these secreted BMP inhibitors. Other BMP antagonists have been identified, and support already exists for direct regulation of BMP ligands and intracellular regulation of Smad proteins by FGF/MAPK signaling (topics discussed later).

More recently, an alternative explanation to the gradient model has been put forth. During gastrulation, a partial or complete inhibition of BMP signaling is adequate to create a “competency zone” to allow other signals (Wnts, FGFs) to specify the NC [31, 53]. Then, BMP signaling must be activated in the NPB at neurula stages to allow the full complement of NPB and NC markers to be expressed. An intriguing new study using *Xenopus* and zebrafish embryos has identified a novel nuclear factor, SNW1, which may mediate this shift in BMP signaling, being responsible for a domain of BMP activity in the prospective NPB at late gastrula stages. SNW1 morphants lack a defined NPB and display clear reductions in early NC markers. Targeted overexpression of *bmp2b* in zebrafish can rescue this phenotype and restore *snail2* expression, suggesting the role of SNW1 in NC development is based on its regulation of BMP activity [52].

Recent experiments from chick embryos also suggest that BMP signals act in two phases and argue against a gradient of activity. Treatment of prospective NC explants from gastrula-stage embryos with Noggin for the first 10 h of culture has no effect on their fate, but treatment after 10 h causes a loss of NC markers and an induction of neural markers. Similarly, treatment of prospective NC explants with Bmp4 after the first 10 h of culture causes no change and crest markers arise normally, but if Bmp4 is applied from the beginning of culture, the explants become prospective epidermis [30]. Together, these findings suggest that neural and epidermal cell fates require continued BMP inhibition or BMP activation, respectively, while the NC is generated by an early phase of inhibition and a late phase of activation. These findings are supported in vivo—Smad1/5/8 signaling is essentially absent during gastrulation, but becomes progressively activated throughout the NPB and NNE by neurula stages, with a sharp drop in activity at the NPB/NP boundary [75, 76]. Also, inhibition of Smad signaling causes a loss of NPB markers [76] and experiments on later-stage (stage 10) chick embryos demonstrate BMP signaling is necessary for the expression of NC specifiers as well [65]. Overall, these studies suggest that BMP/Smad signaling can be completely inhibited at gastrula stages to allow NC induction, but must be activated in the NPB upon neurulation to maintain NC progenitors. This activation of BMP/Smad signaling at neurula stages in the NPB may function to promote the NC fate over the neural fate, as the addition of Bmp4 to NP explants from multiple stages can elicit the expression of Pax7, Snail2, and Sox9 [55, 59, 60, 66, 77, 78].

Although the participation of specific BMP ligands has not been directly addressed in the chick, *Bmp4* expression closely matches the pattern of Smad1/5/8 activation and is postulated to establish the majority of BMP activity at early stages [75]. Expression of *Bmp4* and *Bmp7* becomes enriched in the neural folds and adjacent ectoderm at later stages and exogenous Bmp4 protein can induce NC markers in various contexts in both chick and *Xenopus* tissues [30, 31, 55, 59, 60, 66, 77, 78]. In zebrafish, mutants for both *bmp2b* (*swirl*) and *bmp7* (*snailhouse*) lack NC cells, suggesting a shared role in establishing the necessary domain of BMP activity [71, 79]. Notably, bmp2b in zebrafish is proposed to be functionally equivalent to *Xenopus* Bmp4 [80].

In the mouse, knockouts for *Bmp4*, Type I BMP receptors *Alk2* and *Alk3*, and Type II *Bmpr2* die before or shortly after gastrulation, precluding analysis of NC induction [81–85]. However, heterozygous mutants for Bmp4 show some craniofacial abnormalities, suggestive of a role in NC development [86]. Bmp2-null mutants can survive until E10.5 and lack migrating streams of cranial NC cells and do not develop the first two branchial arches [87, 88]. A follow-up study demonstrates Bmp2 is necessary for migration, but not for induction, since Bmp2-null mice do express early NC markers *Ap2*, *Snail1*, and *Id2* [89]. An epiblast-specific (Mox2-Cre driven) knockout for *Alk3* (*Bmpr1a*) has been generated, and presents expanded anterior neural markers at the expense of surface ectoderm and caudal neural markers. NC markers *Msx1*, *Pax3*, and *Sox10* are still expressed, however, suggesting Alk3 is not necessary for initial NC induction in mouse [90]. Another study uses a Pax3-Cre to conditionally remove Alk3 in the prospective NC and demonstrates that early NC markers are still induced in the cranial region, but NC development in caudal regions is delayed or impaired [91].

A murine line expressing the Cre recombinase from the Wnt1 locus has provided a fruitful tool to analyze later, post-induction events in neural crest development. *Wnt1* is first expressed in the dorsal neural tube, specifically in the NC population at the midbrain region at the four-somite stage shortly before NC migration. Given the earlier expression of *Msx1/2*, *Pax3/7*, and *Ap2*, Wnt1-Cre lines cannot address events leading to the initial induction of NC cells, but are valuable for the analysis of later events. A knockdown of *Alk2*, *Alk3*, or *Alk5* using the Wnt1-Cre causes severe craniofacial, pharyngeal, and cardiac defects, indicating a role for BMP signaling in later NC development [92–95]. Targeted disruption of Smad4 similarly causes multiple craniofacial, pharyngeal, and cardiac anomalies, partially owing to increased levels of apoptosis [96–98]. Although the expression of *Msx1/2*, *Ap2a*, *Pax3*, and *Sox9* at E8.5 is normal in these embryos, expression from E9.5 on is strongly downregulated, implicating Smad signaling in the maintenance of NC markers [98]. Smad4 also participates in Smad2/3 signaling downstream of other TGF-β family members, however, so these phenotypes result from a loss of all Smad signaling. Indeed, Wnt1-Cre mediated *Tgfbr2* knockouts present some of the same defects as those seen in BMP receptor and Smad4 knockouts [99, 100]. Together, these results suggest a later role for BMP/Smad signaling in the mouse, and complement the current models from *Xenopus* and chick that propose a two-phase BMP requirement for NC induction.

### Wnt signaling

Wnts are secreted proteins that initiate a complex cascade of intracellular events, leading to the stabilization of β-catenin in the canonical pathway. Normally, β-catenin is phosphorylated by a complex of proteins including GSK3, APC, and Axin, and then subsequently degraded. Upon binding to cell surface receptors belonging to the Frizzled and LRP families, Wnts cause the activation of Disheveled proteins, which inhibit the β-catenin destruction complex. β-catenin is then free to enter the nucleus where it associates with TCF/LEF family transcription factors to modulate gene expression. Wnt signaling can also activate non-canonical pathways and promote cytoskeletal changes (via Rho-associated kinase in the PCP pathway) or changes in intracellular calcium levels (through activation of PLC and DAG/IP3 signaling). Wnt signaling is implicated in nearly every facet of development and has roles in the generation of multiple organ systems in the embryo. Wnt signaling also maintains a number of adult tissues, and defects in this pathway commonly contribute to cancers (for a review, see [101, 102]).

Wnt signaling has long been associated with NC induction and has recently been proposed to be the inductive signal. In *Xenopus*, overexpression of several different Wnt ligands can induce ectopic NPB and NC marker expression, and Wnts are capable of inducing NC markers in conjugation and animal cap assays, but only when combined with BMP antagonists (Chordin/Noggin) [3, 4, 11, 49, 62–64, 103]. Inhibition of Wnt signaling in *Xenopus* and chick embryos using a variety of extracellular and intracellular modulators has proven the requirement of the canonical β-catenin-mediated pathway for NC induction and later development [3–5, 7, 9, 10, 14, 31, 49, 63, 64, 66, 78, 104–112].

Wnt3a and Wnt8 have emerged as strong canonical candidates to induce the NC in *Xenopus*, being expressed in the DLMZ of the gastrula and at later stages in the caudal NP and paraxial mesoderm, respectively [7, 31, 49]. Specific knockdown of Wnt3a or Wnt8 using morpholinos or a dominant-negative Wnt8 construct inhibits a panel of NPB and NC markers [4, 7, 31, 49]. Interestingly, Wnt3a morphants still express Wnt8, suggesting Wnt3a may act downstream or independently of Wnt8 in NC induction [7]. Wnt8 is also known to be required for zebrafish NC induction. Zebrafish *wnt8* is a bicistronic gene, yielding two transcripts (*wnt8*.*1* and *wnt8.2*), but only morpholinos that interfere with the translation of Wnt8.1 cause a loss of NC markers *pax3*, *foxD3*, and *sox10* [113].

In the chick, *Wnt3a* and *Wnt8a/c* are expressed in the lateral epiblast during blastula stages [114, 115]. At gastrula stages, *Wnt3a* is found in the epiblast and primitive streak along with a number of other Wnt ligands (*Wnt1*, *2b*, *7b*), while *Wnt8a/c* is expressed in the primitive streak and early mesoderm (S. Chapman, personal communication; http://geisha.arizona.edu/geisha). Addition of Wnt3a to prospective neural epiblast explants can eliminate the expression of neural markers and induce the expression of Msx1, Snail2, and HNK-1 (a marker of migratory NC) [30, 114]. Furthermore, inhibition of Wnt signaling in prospective NC explants causes a loss of NC markers, indicating a requirement for ectodermal Wnt signaling in the chick [30]. Although the potential roles of specific Wnts ligands have not been functionally challenged at early stages, Wnt6 has been implicated in later avian NC development. One study suggests signaling from the NNE activates the canonical pathway in the forming neural folds [66] while another proposes Wnt6 induces the NC through the Rho/JNK non-canonical pathway [116]. Much more work is necessary to determine the source and action of the inductive Wnt molecule(s) in avians, particularly at early stages.

In the mouse, *Wnt1* and *Wnt3a* are expressed just before NC migration and participate in later NC development, but do not play a role in the initial induction. Double-homozygous null mutant mice for Wnt1/Wnt3a initially express *Ap2* normally, but expression is lost from the migrating cells. Accordingly, these double-mutant mice have severe abnormalities in NC derivatives [117]. A neural-crest specific (Wnt1-Cre) deletion of β-catenin replicates the midbrain/hindbrain defects of Wnt1 deletion, suggesting Wnt1 signals through the canonical pathway, and additionally presents a near-complete loss of craniofacial structures [118]. Although this β-catenin null mutant makes up for potential canonical Wnt ligand redundancy, the phenotypes may also be the result of compromised cell adhesion. Studies of other Wnt ligands during early murine development do not reveal obvious NC induction defects (summarized, http://www.stanford.edu/group/nusselab/cgi-bin/wnt/mouse).

Additional studies have investigated other components of the Wnt signaling pathway. In *Xenopus*, the Wnt receptor Frizzled7 has been implicated in mediating the initial Wnt signal in the prospective NC domain, with Frizzled3 likely acting at later stages, perhaps responding to Wnt1 signaling [104, 105]. Lrp6, an LDL-receptor related protein, is thought to be a co-receptor for Wnts in NC induction [109], and participates in signaling with a transmembrane protein, Kremen2 [106]. A novel intracellular PDZ domain-containing protein, Kermit, was also shown to be required for NC development, preferentially mediating Frizzled3 signal transduction [119]. Also in *Xenopus*, morpholino knockdown of Disheveled 1 or 2 caused an inhibition of *Snail2* and *Twist*, but depletion of Disheveled 3 had no effect [120]. A recent study using zebrafish and *Xenopus* embryos has identified a novel negative regulator of the canonical Wnt signaling pathway, Kctd15; overexpression of Kctd15 inhibits NC markers, while morpholino depletion causes their expansion. A knockdown of Kctd15 rescues Wnt8.1 morphant zebrafish, suggesting Kctd15 acts downstream of ligand binding to decrease signal strength [121]. Another novel regulator of Wnt signaling is ADAM13, a metalloprotease. ADAM13 was shown to be necessary for cranial NC induction, as morpholinos inhibit expression of *Snail2*, *Sox9*, and *Twist* in *Xenopus tropicalis* embryos. Here, ADAM13 cleavage of Ephrins B1 and B2 is thought to promote Wnt signaling by preventing inhibitory effects of forward EphrinB signaling, thus allowing sufficient Wnt signaling to activate *Snail2* and induce the NC [122]. Non-canonical Wnt signaling may be required for NC induction in *Xenopus* as well—a recent study suggests a crucial role for Wnt11R signaling from the neuroectoderm. The authors demonstrate Wnt11 is capable of activating PAR-1 (also known as microtubule-associated regulatory kinase—MARK) and both molecules are required for early NC markers (*Pax3*, *FoxD3*, *Sox8*), independent of the β-catenin pathway [123].

Interestingly, a recent study using chick explants proposes Wnt signaling mediates the temporal activation of BMP signaling necessary during the second step of NC induction. Treatment of prospective neural explants with Wnt3a at gastrula stages upregulates *Bmp4* expression and can induce NC markers, while treatment of prospective NC explants with a Wnt inhibitor causes a loss of NC markers and a downregulation of *Bmp4* levels [30]. If this same regulation is also present in *Xenopus*, it would explain why animal caps treated with BMP antagonists and Wnt ligands undergo NC induction: BMP antagonists and Wnt ligands promote the early induction of NPB markers, and then the Wnt ligands activate BMP signaling, overpowering BMP antagonism and leading to the expression of NC specifiers.

### Fibroblast growth factor signaling

Fibroblast growth factors (FGFs) comprise a large family of secreted polypeptides (22 genes in vertebrates) that bind to transmembrane receptor tyrosine kinases called FGF receptors (four genes, FGFR1–4 in vertebrates) with the assistance of extracellular matrix components, notably heparin sulfate proteoglycans. Following ligand binding, the receptors dimerize and transphosphorylate one another and activate one or more intracellular signaling cascades, including those mediated by Erk1/2 (MAPK), PKC, and PLC-gamma. Alternative splicing has been reported in several ligands as well as FGFR1–3, adding to the complexity and specificity of ligand/receptor interactions and downstream signaling. FGFs have been implicated in multiple aspects of early development, including mesoderm and endoderm formation, gastrulation movements, anterior-posterior and dorsal–ventral patterning, and neural induction among others (for a review, see [124, 125]).

Several *Xenopus* studies have demonstrated the importance of FGF signaling, with a specific focus on Fgf8a as a NC inducer. This spliceform of Fgf8, unlike Fgf8b, has little to no involvement in mesoderm development [126]. *Fgf8a* is expressed in the DLMZ at gastrula stages, but becomes restricted to more posterior tissues at later stages of development [49, 50]. An Fgf8a morpholino inhibits expression of *Msx1*, *Pax3*, *Hairy2*, *Snail2*, *Sox8/10*, and *Ap2* [3, 4, 11, 49], and unlike Wnts, Fgf8 is capable of transiently inducing NC markers in an animal cap assay without additional BMP inhibitors [50]. Interestingly, overexpression of Fgf8 in low doses expands the expression of numerous NPB and NC specifiers, but in higher doses actually inhibits them [3], suggesting a specific threshold of FGF activity is necessary for NC induction. A recent *Xenopus* study identified a transmembrane protein, Lrig3, which may participate in modulating FGF levels [127]. Lrig3 seems to enhance Wnt signaling, but inhibits Erk1/2 activation and the NC-inducing activity of FGF ligands, potentially though an interaction with FGFR1. Morpholino analysis demonstrates that Lrig3 operates downstream of Pax3 and Zic1 but upstream of NC specifiers Snail2, FoxD3 and Sox9, and could act in the transition from NPB specification to NC specification.

In *Xenopus*, FGFs are likely to act during gastrulation and recent experiments suggest the role of FGF is indirect, acting on mesodermal tissues to induce *Wnt8* expression [49]. The authors show that Fgf8 overexpression is unable to rescue the loss of *Snail2* and *Sox8* in Wnt8 or β-catenin morphants, but overexpression of Wnt8 or β-catenin can rescue NC deficiencies brought about by an Fgf8 morpholino. Furthermore, the combination of Chordin and Fgf8a in an animal cap assay will induce *Pax3*, *Snail2*, and *Sox8*, but addition of a Wnt8 morpholino blocks induction. They also demonstrate that Fgf8a overexpression can expand the domain of *Wnt8* expression and that Fgf8 morphants lack *Wnt8* expression in the mesoderm at late-gastrula stages. A previous study, however, instead suggests that FGF signaling acts directly on the ectoderm; conjugates of DLMZ and animal caps present strong expression of *Snail2*, *FoxD3*, and *Sox9*, but conjugates of DLMZ with animal caps injected with a dominant-negative FGFR1 do not [50]. A similar experiment using a dominant-negative FGFR4a did not cause a loss of NC markers, suggesting that signaling through FGFR1 is a key modulator of NC induction in the ectoderm. In support of a potential ectodermal requirement for FGF signaling, conjugates of neural and epidermal tissue express *Snail2*, but when the neural portion is injected with dominant-negative FGFR1, the conjugates no longer express *Snail2* [61]. Moreover, Wnt3a has been implicated in the ectodermal expression of Meis3, a factor capable of directly activating transcription of *Fgf3* and *Fgf8* in animal cap assays, suggesting FGF may be activated in the ectoderm [7, 9].

A recent study demonstrates an ectodermal requirement for FGF signaling in chick as well [76]. In this work, inhibition of FGF signaling during gastrulation via electroporation of a dominant-negative Fgfr1 or Mkp3 (inhibitor of MAPK signaling) causes a loss of Pax7 and Snail2, but treatment after gastrulation causes no effect. Using epiblast explants and by restricting electroporation to the prospective NPB, this study demonstrates that FGF/MAPK signaling within the gastrula epiblast is required for NC induction. Interestingly, FGF receptors 1 and 4 are expressed in the prospective NC epiblast during this time, but are not found in the mesoderm [76, 128], suggesting FGF does not act on the mesoderm in avian NC induction. FGF signaling is necessary for mesoderm formation [129], however, and many FGF ligands are expressed in the mesoderm and primitive streak (http://geisha.arizona.edu/geisha; 130]). During the stages leading up to gastrulation, Fgf8 is expressed in the hypoblast (the tissue underlying the epiblast), and is thought to participate in neural induction [131], making it an attractive candidate to act on the prospective NC epiblast. Still, the source of the inductive ligand has not yet been identified in the chick, and other FGFs including Fgf3 are expressed in the epiblast itself [132]. The highest level of FGF/MAPK activity during gastrulation is found in the primitive streak [76, 128], and during this and later stages, it is known to regulate the expression of multiple Wnt ligands expressed there, including Wnt3a, Wnt8a/c, and Wnt5b [76, 129, 133]. However, FGF was also found to positively regulate antagonists of the canonical Wnt signaling pathway including NOTUM, Sizzled, Sfrp2, and Cerberus [129]. It would be interesting to determine whether FGF/MAPK regulation of these molecules is necessary for NC induction in addition to its activity within the prospective NC epiblast itself.

A requirement for FGF signaling in zebrafish NC induction has not yet been proven, but FGF/MAPK signaling is crucial for dorsoventral patterning during gastrulation and overexpression of Fgf8 causes an expansion of Ap2 [134, 135]. In the mouse, several FGF molecules are known to act early in development, but have not been functionally linked to NC induction. Null mutants for Fgf4 [136] and Fgfr2 [137] display defects in the inner cell mass, and knockouts for Fgf8 [138, 139] and Fgfr1 [140–142] do not gastrulate properly and thus die before NC formation. Studies have not yet assessed later contributions of these molecules, but all other FGF knockouts generated so far appear to undergo normal NC induction (see [143] for a summary).

In addition to its ability to modulate the Wnt pathway, FGF/MAPK signaling also contributes to BMP antagonism on multiple levels. In *Xenopus* and zebrafish, FGF signaling positively regulates the expression of *Chordin* and *Noggin* during gastrulation [144–146], and negatively regulates BMP ligand expression in the chick and zebrafish [114, 132, 135]. A recent study also shows FGF positively regulates SNW1 in the chick [129], a molecule thought to modulate BMP signaling in *Xenopus* [52]. Additionally, a compelling intracellular regulation of Smad1 has been uncovered, directly linking MAPK signaling to Smad inhibition [147]. MAPK was found to phosphorylate the linker region of Smad1, leading either to Smurf1-mediated polyubiquitination and degradation or exclusion from the nucleus [147–149]. This pathway was shown to be crucial to *Xenopus* neural induction [148, 150, 151], and may operate similarly to cell-autonomously regulate Smad activity in the prospective NPB. Even more intriguing, GSK3, active in the absence of canonical Wnt signaling, was also shown to phosphorylate the Smad1 linker, downstream of MAPK phosphorylations [148]. This provides yet another mechanism whereby Wnt signaling could promote BMP signaling. These findings suggest Smad signaling is a platform to integrate signals from the BMP, Wnt, and FGF pathways. If this is found to be conserved across species, it could account for the reported formation of NC in zebrafish lacking dorsal BMP antagonists (Chordin/Noggin/Follistatin) [53].

### Notch signaling

Notch proteins are transmembrane receptors activated by binding to transmembrane ligands on the surface of adjacent cells. Following ligand binding, multiple cleavage events occur, leading to the intracellular release of the Notch intracellular domain (NICD). The NCID then translocates to the nucleus where it converts the recombining binding protein suppressor of hairless complex from transcriptional repressor to an activator with the help of proteins from the mastermind-like protein family. The NCID is also able to participate in additional transcriptional activation processes, independent from this canonical pathway. Notch signaling has been implicated in numerous developmental processes, particularly involved in establishing boundaries between different cell types (see [152] for a review).

Studies from *Xenopus* demonstrate a crucial role for Notch/Delta signaling, but the precise time of its activity is still uncertain. Glavic et al. show that Notch is expressed in the prospective NC territory, while ligands *Delta* and *Serrate* are expressed in the surrounding regions. They propose that Delta1 interacts with Notch to activate the transcription factor Hairy2, which then suppresses Bmp4 signaling, allowing the inductive phase to proceed [8]. However, this suppression appears to occur during the second maintenance phase, when BMP signaling must be activated. A more recent study using the same tools reports contradictory results, perhaps due to slight differences in the stage of treatment. This group shows that Hairy2 is not regulated by Notch, but is positively regulated by BMP inhibition, the canonical Wnt pathway, and Fgf8, and is downstream of Msx1, Pax3, and Zic1. They propose that Hairy2 maintains NC progenitors, as overexpression represses NC markers and upregulates NPB markers [11]. A follow-up study finds that Hairy2 actually activates the Notch pathway cell-autonomously, activating Delta1 via STAT3. Delta1 then acts non-cell-autonomously to upregulate *Id3*, *Snail2*, and *Sox9* [12]. This suggests Hairy2 may act as a trigger for Notch/Delta signaling at the maintenance phase, eventually leading to the expression of NC specifiers. Another *Xenopus* study extensively characterizes a novel secreted protein, Tsukushi (Tsk), necessary for NC induction. Tsk is capable of acting as a BMP antagonist through direct binding to Bmp4, while also regulating the Notch pathway by binding to the extracellular domain of Delta1. The authors propose Tsk is essential to establish the proper level of BMP signaling at the prospective NPB during gastrulation, together with Notch signaling [153]. Potentially, Hairy2 and Tsk serve to modulate Notch and BMP signaling at multiple levels of NC development, but more research is needed.

In the chick, one study has assessed Notch signaling, demonstrating a role in refining the NC domain after induction has taken place. In this instance, Notch seems to act indirectly. Both overactivation and inhibition of Notch signaling cause an inhibition of *Bmp4* in the epidermis and *Snail2* in the neural fold, but overexpression of Bmp4 in these embryos can rescue the loss of *Snail2* expression. This suggests Notch acts primarily to regulate *Bmp4* levels [154]. Notch has yet to be implicated in NPB specification during gastrulation in chicks.

Notch signaling has also been linked to NC development in zebrafish embryos, though it seems to act primarily by restricting the neural domain. A loss of Notch/Delta signaling in *mindbomb* (*mib*) mutant zebrafish causes a loss of NC derivatives at the expense of lateral NP derivatives such as interneurons [155]. Another study suggests Notch acts via repression of Neurogenin-1 function, restricting neurogenesis without actively promoting NC formation [156]. More recent studies suggest Notch/Delta acts earlier, refining the border of the neural plate specifically through negative regulation of the transcription factor *prdm1a* (*Blimp1*) [157]. Prdm1a, necessary for NPB specification in zebrafish, antagonizes another factor olig4, which defines the lateral edge of the NP and promotes neural cell fates over NC [23, 157, 158]. It appears olig4 is restricted by BMP signals during gastrulation, as *swirl/bmp2b* mutant zebrafish demonstrate a laterally expanded expression of olig4 [158]. Importantly, in all these zebrafish studies, inhibition or loss of Notch signaling primarily affected trunk, but not cranial NC cells, suggesting it is not responsible for the initial induction of all NC cells.

Mouse mutants for members of the Notch signaling pathway generally display an increase in neuronal differentiation markers and a decrease in progenitor markers, demonstrating a critical role in the early stages of CNS development (for a review, see [159]), but there is no support for a role in NC induction. Homozygous null mutants for Delta1 display proper generation of NC cells, but show defects in migration and differentiation [160], consistent with a later role for Notch signaling.

### Other signaling pathways

Retinoic acid (RA) signaling has an established role in caudal neural patterning, and may act in NC development as well, though it seems to act after the initial induction. RA signaling is restricted to the caudal portions of the embryo during early development, owing to the posterior localization of the RA-synthesizing enzymes (retinaldehyde dehydrogenases, Raldhs) and the anterior localization of RA-degrading enzymes (Cyp26 family members). A study using *Xenopus* animal caps showed that induction of *Pax3* by chick mesoderm or NP does not require RA [161] suggesting RA is not necessary for NC induction. Another study using *Xenopus* embryos demonstrated that addition of exogenous RA or over-activation of RA signaling can expand *Snail2* expression anteriorly, whereas treatment with a dominant-negative RA receptor causes a posterior expansion [110], suggesting RA effects on the NC are secondary to axial patterning. An avian study using vitamin A-deficient (VAD) quails (which lack the RA precursor) suggests RA is required for the survival of migrating NC cells; VAD quails appear to form cranial NC cells properly, but within a few hours of migration, they undergo extensive apoptosis [162]. A mouse study evaluated RA signaling in the cranial neural crest and provides a slightly different perspective [163]. As a result of knocking out both Cyp26a1 and c1 together, RA signaling is expanded anteriorly, and although NC markers *Snail* and *Sox9* are expressed normally in the cranial neural folds, migrating NC cells are largely absent. Interestingly, crossing Cy26a1/c1 double mutants with a null mutant for Raldh2 (the only RA-synthesizing enzyme present at these stages) rescues the NC migration defect, despite the expected absence of RA signaling in these embryos. This suggests that although over-activation of RA signaling can disrupt cranial NC migration, endogenous RA signaling is not required for migration. Furthermore, because NC markers were initially expressed normally, RA signaling does not appear to function early in NC induction in the mouse.

A recent study evaluates the role of Indian Hedgehog (Ihh) in early neural crest development in *Xenopus* [164]. Here, the authors demonstrate Ihh signaling is necessary for NC induction, maintenance of progenitors; loss of Ihh function using morpholinos, dominant-negative constructs, and chemical inhibition causes a loss of both NPB and NC specifiers and an expansion of neural and epidermal markers. They reveal requirements for autocrine signaling within the prospective NC and paracrine signaling from the mesoderm, with mesoderm-based signaling further being necessary for proper migration. A well-characterized member of the hedgehog family, Sonic hedgehog (Shh), has established roles in the formation of left/right asymmetry and ventral patterning of the spinal cord, and although ectopic application of Shh can repress NC markers at later stages in the chick [59, 65, 165], an endogenous role in NC induction has yet to be proven.

Lastly, Endothelin signaling has also been implicated in *Xenopus* NC induction [32]. Using a combination of in vivo and explant approaches, the authors suggest Endothelin-1, released from the mesoderm, functions in both NC specification and cell survival. They demonstrate that morpholino knockdown of the Endothelin-A receptor and chemical inhibition of Endothelin signaling causes a loss of NC specifiers *FoxD3*, *Sox9* and *Sox10*, but not the NPB specifier *Msx1*, suggesting a role in NC progenitor maintenance during the mid-neurula stage. With NC induction requiring the precise temporal regulation of multiple transcription factors, additional signaling pathways are surely yet to be uncovered.

## Perspectives

### Posteriorization and early inducers

Wnts, FGFs, and retinoic acid (RA) are proposed to act as caudalizing or posteriorizing factors during early development. In neural development, it is thought that the NP is initially composed entirely of rostral or anterior character, subsequently being posteriorized by the action of Wnts, FGFs, and RA to give rise to caudal components of the nervous system (reviewed in [166, 167]). Since these same factors are involved in NC induction, it was proposed that the NC is a result of NPB posteriorization [110]. A study in chick, however, indicates the prospective NPB is initially specified by gastrula stages to become NC at all axial levels, requiring the inhibition of Wnt signals at later stages to allow placodal development anteriorly [30]. Additionally, work in *Xenopus* suggests Wnt signaling acts directly to induce the NC, independent of its role in antero-posterior neural patterning [111]. Recent *Xenopus* studies identify two factors, Gbx2 and Meis3, as direct targets of Wnt/β-catenin signaling necessary for NC induction [7, 10]. Although both of these genes are involved in caudal neural patterning, the NC-inducing activity of Gbx2 is separable from its role in NP posteriorization [10]. Gbx2 is also shown to cooperate with Zic1 to specify the NC fate, while Zic1 activity alone leads to placodal development [3, 10]. These findings argue that NPB antero-posterior patterning and NC induction are distinct from events in early neural development.

Although these studies describe Gbx2 and Meis3 as some of the earliest-expressed proteins necessary for NC induction and direct targets of Wnt signaling, another recent study proposes Ap2a is the earliest-acting factor in *Xenopus* [6]. *Ap2a* is broadly expressed, including the prospective NPB from the onset of gastrulation (stage 10) and preceding the expression of all other NPB specifiers but still downstream of Wnt/β-catenin signaling. They demonstrate that morpholino depletion of Ap2a causes a loss of Msx1, Hairy2, Pax3, and Snail2, and a gain-of-function upregulates them. Although the initial expression of Ap2a is not affected by morpholinos against other NPB specifiers, later expression is. A zebrafish study also suggests Ap2a (Tfap2a) is a critical early regulator of the NC fate, along with FoxD3 [21]. These two factors are expressed during gastrulation and are necessary and sufficient for NC development. Interestingly, double-mutants of Ap2a and FoxD3 actually present altered patterns of BMP and Wnt signaling, suggesting these factors participate in establishing the signaling environment required for NC induction. The expression patterns of Ap2 and FoxD3 in the chick, however, are not consistent with an early role in NC induction [16]. Instead, Pax7 is currently the earliest-expressed factor necessary for NC induction in the chick [14]. This marker is expressed exclusively in the NPB soon after gastrulation and later labels the entire neural folds. This contrasts with other NPB specifiers that are found more lateral (Msx1, Pax3) and NC specifiers, which are initially only expressed in the cranial NC (Snail2, FoxD3, Sox9) [16].

### Neural and neural crest induction: shared first steps?

Recent studies in the chick and frog describe the initial induction of the NC taking place during gastrulation, requiring the activation of FGF and Wnt signaling and the inhibition of BMP signaling. Studies from these same organisms suggest neural induction also requires BMP inhibition and the activation of FGF/Erk signaling from the blastula to the gastrula stage [114, 131, 132, 168–170] (reviewed by [171]), prompting the question of whether neural and NC cells are initially specified together.

In the chick, Erk1/2 proteins are activated throughout the epiblast, encompassing both the prospective NP and prospective NPB from blastula to gastrula stages [76, 128]. Although studies of NC induction have not analyzed effects of FGF/Erk inhibition at pre-gastrula stages, inhibition at gastrula stages causes a loss of NPB markers in their endogenous domain, but an upregulation in the NP [76]. Thus, if FGF is to act similarly on these populations, it likely does so before gastrulation. Furthermore, activation or inhibition of Wnt signaling can interchange the expression of neural and NC markers in explants taken at pre-gastrula [30, 114] but not post-gastrula stages [55], suggesting Wnts mediate the initial choice between these fates before gastrulation. Indeed, Wnt ligand expression and nuclear β-catenin are found in the lateral epiblast, but absent from the medial, neural-specified epiblast at blastula stages [114, 115, 172]. Furthermore, in *Xenopus*, inhibition of Wnt signaling causes a strong expansion of neural plate markers at the expense of NC and placodal markers. Yet, upregulation of canonical Wnt signaling interferes with neural induction, and this activity can be separated from Wnt-mediated NC induction [107].

In *Xenopus* animal cap assays, addition of BMP antagonists such as Chordin or Noggin is sufficient to induce neural markers (the animal caps are said to be “neuralized”). To launch and maintain NC markers in this assay, caps must first be neuralized and then treated with FGF or Wnt agonists, supporting a potential shared requirement for BMP antagonism. However, BMP antagonism seems to behave differently toward markers of neural and NC; inhibition of BMP signaling can induce ectopic neural and NC markers prior to the blastula stage, but inhibition at early gastrula stages only generates ectopic NC [173]. Similarly, inhibition of BMP signaling in lateral ectoderm of the chick at gastrula stages can induce ectopic NPB and NC markers [174], but not neural markers [169]. Interestingly, BMP ligands are expressed throughout the epiblast at blastula stages [132, 175], despite the seeming requirement for BMP inhibition to initially promote both neural and NC fates. Perhaps signaling at the blastula stage in chick is primarily utilized to maintain the pluripotency of the epiblast, similar to the situation in the mouse (discussed below).

### Early signaling insights from mouse studies

Although the BMP, Wnt, and FGF signaling pathways are needed to establish cell fates at gastrula and neurula stages, studies of mouse embryonic stem (ES) cells (derived from the inner cell mass of the blastocyst) and epiblast stem cells (EpiSCs) suggest dynamic and temporally segregated roles earlier in development. The FGF/Erk signaling cascade must be inhibited to allow self-renewal and pluripotency in mouse ES cells, with activation of the pathway driving them towards differentiation (summarized in [176]). Along with Erk inhibition, GSK inhibitors are required to retain a pluripotent state, but this activity seems to be independent of the Wnt pathway. Alternatively, leukemia inhibitory factor (LIF) and Bmp4 are capable of maintaining mouse ES cells in an undifferentiated state. Autocrine Fgf4/Erk signaling appears to push mouse ES cells towards differentiation [177] and Erk signaling seems to be necessary for their adoption of the neural fate [170], but an in vivo study of mouse development suggests FGF signaling is not necessary for neural induction [178]. Studies of earlier development support an endogenous role for BMP signaling [178] and FGF/Erk inhibition [179] in maintaining pluripotency of intact embryos, suggesting the initial differentiation activity of FGF may be sufficient to establish the competence of the epiblast for neural development. In support, the addition of exogenous FGF ligands is essential for the self-renewal and pluripotency of EpiSCs (derived from the gastrula epiblast), with inhibition of the FGF/Erk signaling leading to differentiation [180]. This is in stark contrast to mouse ES cells where FGF/Erk signaling provides the opposite instructions. Indeed, FGF signaling in EpiSCs actually serves to inhibit neural induction [181]. Together with ES cell studies, these findings suggest FGF has an initial role in epiblast formation, but then serves to maintain pluripotency of the epiblast [182]. These changing roles of FGF signaling caution us to consider temporal differences in cellular responses and underscore the dynamic nature of signaling pathways.

### Human neural crest development

Advancing our limited understanding of human NC development will surely improve our capacity to address the many human maladies associated with improper NC development. Model organisms have provided invaluable information on NC development and while many molecules and processes are conserved (as described above), the deviations that are present make it imperative to specifically study human NC biology. The morphology of early human NC development has been depicted from careful histological analysis [183]. An extensive molecular profiling study was performed on cell lines derived from human neural tube explants, presumed to be NC, and indicates the human NC shares many markers with stem cells. Additionally, the study suggests conservation among NC cells of the chick and mouse, but also points to unique traits in the human NC [184]. More recently, a battery of NC markers were analyzed in cranial and trunk regions of intact early human embryos (Carnegie stages 12–18), confirming a broad conservation of expression profiles with model organisms [185]. This study identified Pax3, Sox9, and Sox10 expression in pre-migratory NC at early stages of caudal trunk development with Ap2, Pax7, Sox9, and Sox10 expressed during early migration at more anterior locations. Of note, HNK-1 identified few migratory NC and while p75 recognized many more, it only labeled a subset of NC cells. More importantly, p75 also labeled many non-NC cells. This is particularly relevant, given the broad use of these two markers amongst stem cell biologists (see below).

Given the obvious experimental limitations and restrictions of studying early human development, human embryonic stem cells (hESCs) provide an excellent alternative to study the human NC. While it is difficult to argue how close this system replicates embryonic development, it does have the capability to challenge the potential of cells in many different contexts and to expose used mechanisms and restrictions. hESCs can be induced to form NC-like cells capable of differentiating into nearly all known NC derivatives. These progenitor cells have been generated by several different protocols in varying culture conditions [186–198]. Initial work relied on stromal cell co-cultures [186, 188, 191], and later progressed to derivation from embryoid bodies [187, 198] or neurospheres [192, 193, 196]. Cleaner and more efficient protocols have been recently reported to derive NC from hESCs in cultures of adherent cells in serum-free defined media, without complex intermediary structures [190, 194, 197].

Culture density of hESC was reported to alter neural and NC formation in a differentiation protocol including FGF2, Insulin, and gradual exposure to both BMP and Nodal/Activin inhibition (Noggin and SB431542) [190, 194]. Another study demonstrated that both BMP and Wnt signaling were necessary for NC markers in cells derived from neural rosettes, but it was unclear whether these signals were required for the initial marker expression, or for their maintenance and the generation of derivatives [193]. In several of these pioneering studies, HNK-1 and p75 were used to screen for NC progenitors, but recent findings highlight complications in the use of these markers. In the human, p75 marks only subsets of NC and additionally labels many non-NC cells, while HNK-1 labels a smaller fraction of NC [185], and in at least one study, their use was shown ineffective to discriminate between NC and non-NC [193]. Still, HNK-1-positive, p75-positive cells induced from hESCs do exhibit the capacity to generate an array of derivatives characteristic of the NC. In some of these studies, NC-like cells arise from Pax6+ neural precursors, while in others, an alternative origin has been proposed. Yet, no studies have addressed their possible equivalence or distinct differentiation potential. An intriguing recent study reports a direct protocol for the generation of NC progenitors from hESCs in 12–14 days using a GSK3-β inhibitor (BIO) to activate canonical Wnt signaling, Noggin to inhibit Smad1/5/8 signaling, and SB431542 to inhibit Smad2/3 signaling [197]. Interestingly, removal of Noggin has no effect, perhaps owing to the low level of basal Smad1/5/8 activation, but addition of BMP4 suppresses the generation of these cells, suggesting high levels of BMP/Smad signaling are not conducive to the formation of NC-like cells from hESCs. These conditions mimic some of those currently thought to induce the NC in the model organisms studied, and underscore the value of continued research in NC induction. Reciprocally, the study of hESC biology will undoubtedly unveil exciting new insights into the signaling events in NC development in vivo.

## Summary of inductive signals

**Fig. 3:**
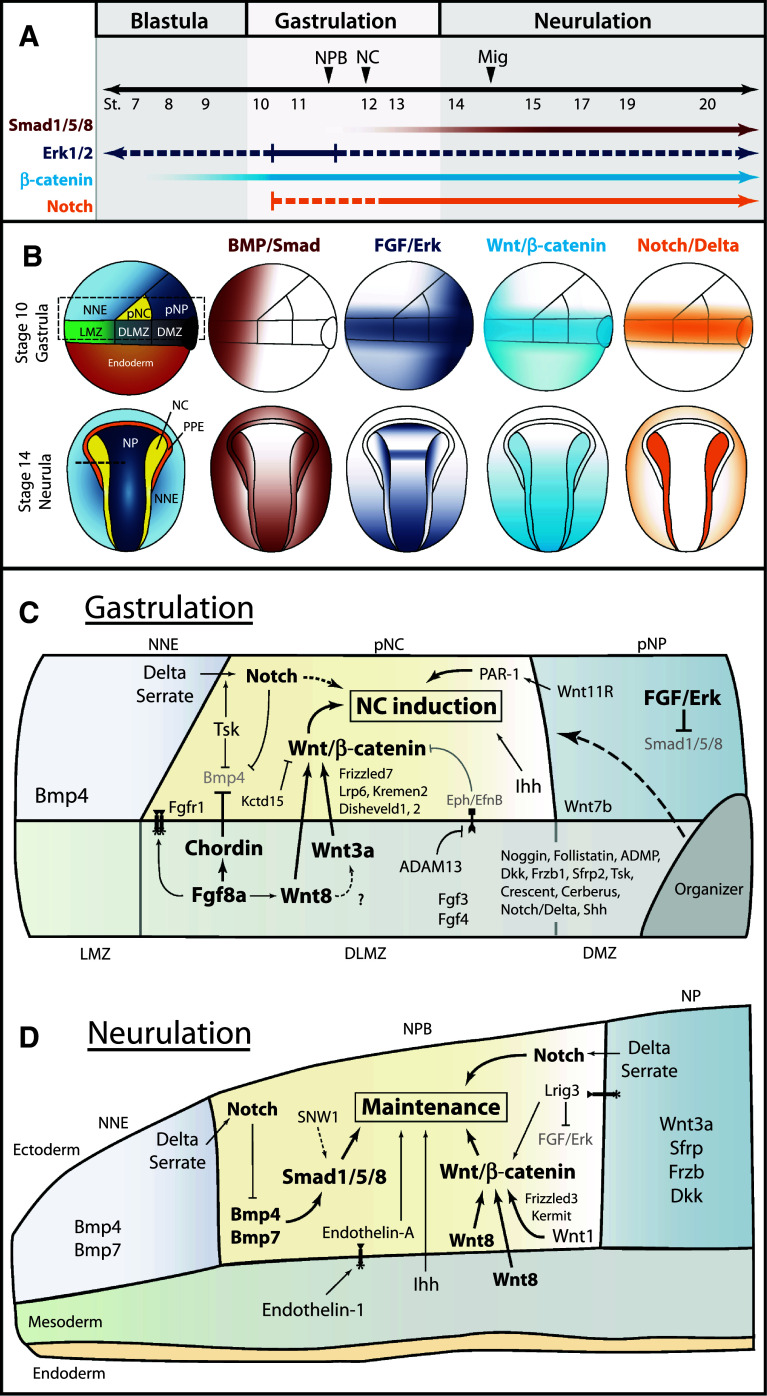
Temporal and spatial participation of signaling molecules involved in *Xenopus* neural crest induction. **a** Timeline of signaling pathway activation and requirement in early NC development.* Closed arrows*/*lines* indicate activation and requirement in NC tissues. *Dotted lines* indicate activation in NC tissues, but requirement is unknown. BMP/Smad signaling must be inhibited during gastrulation, but activated upon neurulation. The specific stage when Smad signaling first becomes activated in the NC has not been determined. FGF/Erk signaling is activated throughout early NC development, but has only been functionally implicated during early gastrula stages [49, 50]. Wnt/β-catenin signaling is thought to be required at all stages of early NC development, but becomes more strongly activated by neurulation [31]. The precise time when Notch is required is still debated, but may play a role in the initial induction during gastrulation. **b** Spatial activation of BMP, FGF, Wnt, and Notch signaling during *Xenopus* gastrulation and neurulation. BMP/Smad, FGF/Erk, and Wnt/β-catenin activation based on data from [199, 200]. Notch/Delta activation inferred from requirements in germ layer segregation and NC development [8, 12, 201]. Overall, spatiotemporal activation of these pathways is conserved between *Xenopus* and zebrafish. **c** Spatial expression and participation of signaling molecules in *Xenopus* neural crest induction at the gastrula stage. Diagram corresponds to *dotted box* of stage 10 gastrula in **b**. Molecules in bold have support from multiple studies. *Solid lines* indicate known relationships, *dotted lines* indicate potential relationships. NC induction results from the combined action of Wnt/β-catenin, FGF, Indian Hedgehog, and non-canonical Wnt signaling. Fgf8a is thought to regulate the expression of Wnt8 in the dorsolateral marginal zone (DLMZ), but may signal to the prospective neural crest itself. Wnt8 and Wnt3a signaling from the DLMZ activate canonical Wnt signaling in the prospective neural crest. Multiple agonists and antagonists of BMP and Wnt signaling are expressed in the Organizer, DMZ, and DLMZ and function in dorsal–ventral and anterior–posterior patterning, and these molecules likely also participate in NC induction (*dotted line*). Expression of other potential signaling molecules and regulators is presented. See main text for details on the participation of individual signaling molecules. **d** Participation of signaling molecules in the maintenance of NC progenitors in *Xenopus* neurulation. Diagram corresponds to section at *dotted line* in stage 14 neurula in **b**. NC maintenance requires activation of Smad1/5/8, Wnt/β-catenin, Notch/Delta, Indian Hedgehog, and Endothelin-A signaling. BMP and Wnt signals are likely mediated by Bmp4, Bmp7, Wnt1, and Wnt8, expressed in the neural folds upon neurulation. Additionally, Wnt8 is present in the paraxial mesoderm and Wnt3a in the neural plate. Notch signaling is thought to operate both by regulating Bmp ligand levels and leading to the expression of NC specifiers. See main text for details on the participation of individual signaling molecules. Expression data gathered from references in text and from http://www.xenbase.org

**Fig. 4:**
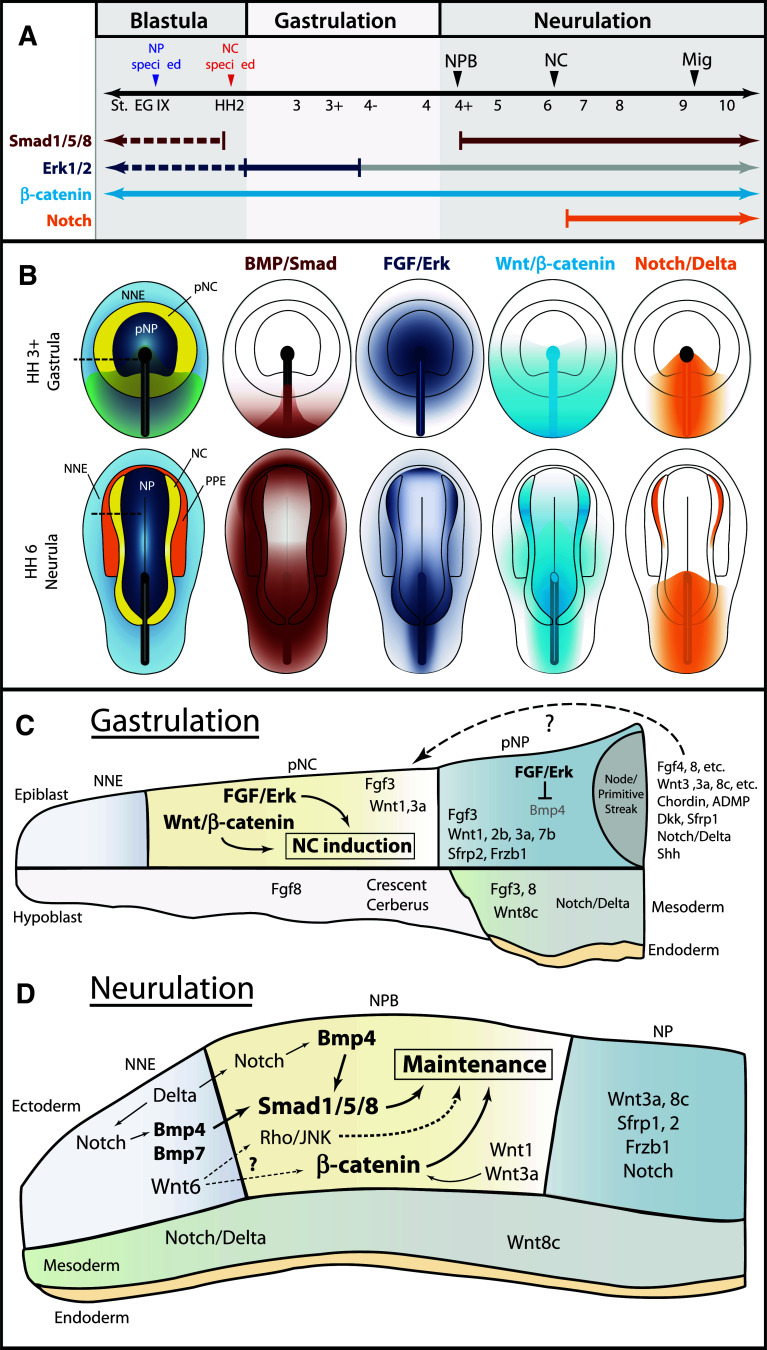
Temporal and spatial participation of signaling pathways involved in chick neural crest induction. **a** Timeline of signaling pathway activation and requirement in early NC development. *Closed arrows/lines* indicate activation and requirement in NC tissues. *Dotted lines* indicate activation in NC tissues, but requirement is unknown. Smad1/5/8 signaling is active in the entire epiblast at blastula stages, but is inactivated by gastrulation [75]. Signaling becomes active with the expression of NPB markers, and remains active through to migration. Erk signaling is also active in most of the epiblast at blastula stages, and is required for neural induction until gastrulation. A requirement for FGF/Erk signaling in NC induction was only demonstrated during gastrulation. Erk signaling remains active in the NPB and NC tissues through to migration, but is no longer required for NC development (*gray line*). Wnt/β-catenin signaling is thought to be necessary for all stages of early NC development. A requirement for Notch/Delta signaling was demonstrated at mid-neurula stages. **b** Spatial activation of BMP, FGF, Wnt, and Notch signaling during chick gastrulation and neurulation. BMP/Smad activation based on [76]. FGF/Erk activation based on [76, 128]. Wnt/β-catenin activation inferred from expression of agonists and antagonists, and functional requirements for Wnt signaling. Notch/Delta activation is based on expression of molecules and functional requirements [154]. **c** Spatial expression of relevant signaling molecules and requirements for chick neural crest induction during gastrulation. Diagram corresponds to section at *dotted line* HH 3+ gastrula in **b**. Functional studies have demonstrated a requirement for FGF/Erk and Wnt/β-catenin signaling, but the participation of specific signaling molecules has not been challenged. The spatial expression of some potential signaling molecules is presented. Multiple FGF and Wnt agonists and BMP and Wnt antagonists are expressed in the node/primitive streak, but it is unclear whether these molecules can diffuse the distance to influence the prospective NC tissue (*dotted arrow*). **d** Spatial expression and participation of signaling molecules and pathways in the maintenance of NC progenitors during chick neurulation. Diagram corresponds to section at* dotted line* in HH 6 neurula in **b**. Smad1/5/8 and β-catenin are likely activated by Bmp4, Bmp7, Wnt1, and Wnt3a expressed in the neural folds and adjacent NNE. Wnt6 in the NNE has also been implicated in NC development, but may act through the non-canonical Rho/JNK pathway. Notch signaling likely participates indirectly by regulating Bmp4 expression. Spatial expression of other potential signaling molecules is presented. Expression data gathered from references in the text and from http://geisha.arizona.edu/geisha

**Fig. 5:**
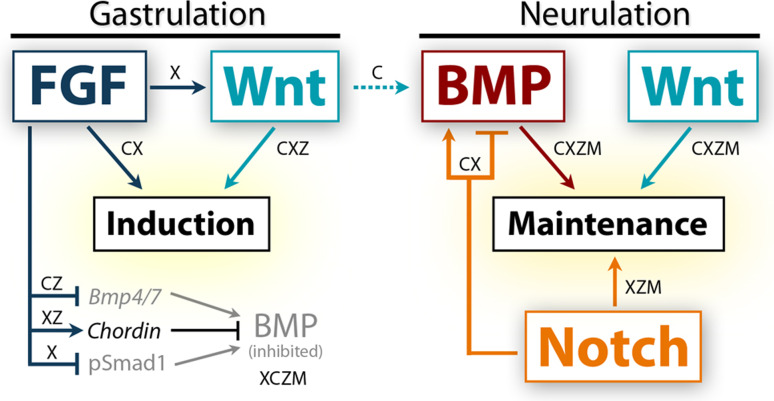
A model of signaling participation during the two phases of neural crest induction. In this figure, we present a simplified model of the major signaling events thought to occur in NC induction, drawing on evidence from all four of the organisms discussed. Since the precise time, source, and cross-regulation between pathways vary between species, the model organism is noted where a specific interaction or activity is known to occur. *X* *Xenopus*, *C* chick, *Z* zebrafish, *M* mouse. During gastrulation, FGF and Wnt signaling are both known to induce the neural crest at the prospective NPB, activating the expression of NPB specifiers. *Xenopus* studies demonstrate that FGF regulates Wnt signaling during this first phase, but evidence from chick and *Xenopus* suggests FGF acts directly as well. BMP signaling must be at least partially inhibited for this first step, and FGF participates in BMP attenuation on multiple levels. The transition to the second phase involves the activation of BMP signaling, and research on chick explants suggests Wnts may participate in this BMP activation. Since FGF contributes to BMP antagonism before and during gastrulation, the restriction of FGF activity or insensitivity of the NPB to FGF signals also likely plays a role in this transition. In the second phase, BMP and Wnt signaling converge to maintain the expression of NPB specifiers and initiate the expression of NC specifiers. Notch signaling is known to refine the domain of BMP activity, but some evidence suggests Notch acts directly on the neural crest population as well. Throughout later neural crest development, these signaling pathways continue to participate in migration and differentiation

As we learn more about the molecular events leading to the specification of the neural crest, we unveil subtleties in the induction mechanism employed by each organism. Graphical summaries of the spatiotemporal activation of signaling pathways and participation of signaling molecules are presented for the two most extensively studied organisms, *Xenopus* (Fig. 3) and chick (Fig. 4). Taking the information from these two models and integrating findings from zebrafish and mouse, an overall theme of neural crest induction has emerged. The initial induction of the neural plate border appears to involve signaling events from the blastula to the gastrula stage, with continued signaling taking place during neurulation to maintain neural crest progenitors and bring about the expression of neural crest specifiers. In this model, FGF and Wnt signaling are required for the initial phase of neural crest induction with Wnt, BMP, and Notch signals acting later to maintain neural crest development. A simplified model is presented in Fig. 5.

The first phase requires at least a partial attenuation of the BMP/Smad signaling pathway, but by neurula stages BMP signaling must be activated. BMP inhibition is crucial for both neural and neural plate border specification, and might be regarded as a required step in establishing the competency of the prospective ectoderm. BMP attenuation is likely achieved though multiple redundant methods, including the limited expression of BMP ligands, the activity of secreted BMP antagonists (Chordin, Noggin, and others), and FGF/MAPK-mediated intracellular Smad inhibition. The activation of BMP signaling at neurula stages may be temporally regulated by Wnt signaling. Additionally, the canonical Wnt signaling pathway must be activated within neural crest progenitors themselves and throughout early neural crest development. Analogous to other developmental processes, Wnt signaling is capable of mediating cell-fate decisions, and likely directs the choice between neural and neural crest progenitors. Notch also promotes neural crest development over neural cell fates at multiple points in development, but the precise mechanism(s) remain to be understood. Lastly, a functional FGF signaling pathway is necessary for the initial induction of neural plate border specifiers. However, FGFs are capable of regulating Wnt and BMP signaling and are crucial for several other developmental events (including neural induction, mesoderm development, gastrulation movements, and early epiblast pluripotency and competence), all of which could influence NC induction. In the years to come, the real challenge will be to understand the cross-regulation and combinatorial inputs of these and other signaling pathways, their precise temporal effects, and how they integrate to establish the neural crest program. Since recent studies have found that the NC is specified at pre-gastrula stages, a more thorough analysis of these signaling pathways is called for at early stages of development to understand the complete transcriptional cascade of events that enable the amazing plasticity of the neural crest.

## Abbreviations

NC: Neural crest
NPB: Neural plate border
NP: Neural plate
NNE: Non-neural ectoderm
pNC: Prospective neural crest
pNP: Prospective neural plate
DLMZ: Dorsolateral marginal zone
ES: Embryonic stem
EpiSC: Epiblast stem cell
hESCs: Human embryonic stem cells
